# Hemodiafiltration Should Be the Primary In-Center Kidney Replacement Modality in ESKD: Commentary

**DOI:** 10.34067/KID.0000000847

**Published:** 2025-06-11

**Authors:** Thomas A. Golper

**Affiliations:** Vanderbilt University Medical Center, Nashville, Tennessee and Larner College of Medicine, University of Vermont, Burlington, Vermont

**Keywords:** ESKD, renal replacement therapy

Bernard Canaud and I met in Montpellier, France, in 1990 as he and Charles Mion were essentially creating online hemodiafiltration (HDF). I was influenced by Karl Koch, Stanley Shaldon, Charles Dinarello, Nathan Levin, Dick Ward, Alfred Cheung, and others regarding the effect of water quality on hemodialysis outcomes, and the Montpellier approach regarding dialysis water quality was novel. The dialysis facility Medical Director is responsible for the quality of the dialysis (*e.g*., water quality) but also must be a wise steward of resources such as dialyzer reuse, staffing ratios, conversion from high-flux hemodialysis to high volume HDF (HVHDF, which means convective volumes per session of ≥23 L). Thus, the quality and cost of dialysis-like care has been my interest for decades and influences these remarks. As my third commentary in high impact journals regarding HDF in the United States,^1,2^ I am attempting a point-counterpoint approach to the positions taken by Canaud versus Sharma and Lin.^3,4^

## Outcomes and Costs

Canaud mentions that 3–4-hour conventional hemodialysis sessions are associated with inferior outcomes; hence, we need improvement, and HVHDF may be a solution.^3^ I counter with the simple argument that we should strive to perform at least 4-hour hemodialysis sessions to achieve two goals: slower ultrafiltration rates and greater removal of smaller middle molecules. Longer treatments decrease patient satisfaction, require more staff time, and increase facility expenses. However, no new equipment or techniques are needed. Is that an acceptable compromise?

Sharma and Lin request a study of HVHDF in diverse populations before its broad implementation.^4^ I think that would be a waste of limited resources (see below). The findings of Dialysis Outcomes and Practice Patterns Study, an observational study with limited diversity, suggest that patients exposed to longer duration hemodialysis sessions (versus shorter) have better outcomes.^5^ Canaud cites the patient-reported outcome measures data of the Comparison of High-Dose Hemodiafiltration with High-Flux Hemodialysis (CONVINCE) study that shows that in several domains, HVHDF was superior to high-flux hemodialysis.^6^ The suggestion to increase time on high-flux hemodialysis and patient dissatisfaction with that must be addressed in the context of the CONVINCE study patient-reported outcome measures results. This looks to favor HVHDF.

Cost-effectiveness was argued in the context of water usage.^7^ These are the only data suggesting that less water is used in HDF. Hemodialysis does require a lot of water, but the quantity of water used as dialysate and/or substitution fluid is far less than reverse osmosis (RO) rejected water necessary for either hemodialysis or HDF. In the United States, I am not aware of any jurisdiction that allows RO rejected water to re-enter the community's processed water system. That is a larger “waste” of water than that needed for dialysate or substitution fluid. The spent dialysate goes down a similar drain as the rejected RO water. Both HVHDF and hemodialysis will have RO rejected water in similar amounts that will not be locally recycled.^8^

## Implementation Barriers

By April 2025, Fresenius had not yet determined the cost of conversion to HVHDF from high-flux hemodialysis in its US facilities. This is a complex process of balancing the investment costs of converting to the 5008X machines and the FX CorAL dialyzers versus the fixed payment from Medicare. Furthermore, the growth of Medicare Advantage plans onto the dialysis sector increases uncertainty for facility payments. The facility payment from commercial insurers is much greater than that of Medicare or Medicare Advantage but is also declining. Hence, concurrent financial pressures influence the potential movement toward HVHDF.

## Other Issues

Sharma and Lin are impressed with the benefits of HVHDF but advocate only a limited adoption. They think that sicker patients may not tolerate HVHDF and thus not derive the same benefits as healthier patients. I disagree as I mentioned previously.^2^ All prospective HDF randomized controlled trials used prevalent patients to shorten the recruitment period. However, those are the patients more likely to be sicker than incident dialysis patients because they suffered the ravages of uremia longer. If their rate of progression was short, it was because of some aggressive systemic disease. Most end-stage patients got there through a slow progression. Studying prevalent dialysis patients selects patients likelier to be sicker than incident patients. I propose that the greatest benefit of HVHDF will be in incident patients where better depuration occurs from the onset of dialysis dependence.

Regarding the Sharma and Lin concerns about diversity, one could take this to extremes. The countries providing patient outcomes with HVHDF have a paucity of African descendants. Should we divert resources to study the outcome of HVHDF on subsets of diverse populations or use those resources to convert to HVHDF where possible and then observe results? We would compare HVHDF versus ≥4 hours of high-flux hemodialysis or versus expanded hemodialysis using medium cutoff membranes (see below). We can all think of studies we would like to see. At some point, limited resources argue to get on with what you think is the best overall strategy.

The UK High-Volume Hemodiafiltration vs. High-Flux Hemodialysis Registry Trial underway in HVHDF experienced centers will provide granular comorbidity and cost data.^9^ The other imperative outcome study involves the removal of small middle molecules using medium cutoff membranes where the sizes of toxins removed by diffusion and convection approximates the removal seen in HVHDF.^10^ The experience with medium cutoff membranes dialyzers is much less than that of HVHDF, but the initial conversion cost is considerably lower. In polling the chief medical officers of many dialysis organizations, the outcomes of these studies will be impactful in their decisions to implement HVHDF. We will know a lot more in a few years.

## Supplementary Material
