# Supplementary material for: Hemodiafiltration Should Be the Primary In-Center Kidney Replacement Modality in ESKD: Commentary
Source: Kidney360. 2025 Jun 11;6(10):1655–6. doi: 10.34067/KID.0000000847 (PMC12778009; doi:10.34067/KID.0000000847)
Supplement: Supplementary file 1 [file kidney360-6-1655-s001.pdf]

ASN American Society of Nephrology
JASN Journal of the American Society of Nephrology
KIDNEY360 Accessing Our World From Every Angle
CJASN Clinical Journal of the American Society of Nephrology

# ASN Journal Disclosure Form

As per ASN journal policy, I have disclosed any financial relationships or commitments I have held in the past 36 months as included below. I have listed my Current Employer below to indicate there is a relationship requiring disclosure. If no relationship exists, my Current Employer is not listed.

T. Golper reports the following:
Employer: Robert Larner College of Medicine at the University of Vermont; Consultancy: NxStage; Lightline; Exterius, Medical Education Institute; Research Funding: Renal Research Institute; Honoraria: Up To Date; Home Dialysis University, NephroNet,; Patents or Royalties: Up to Date; Advisory or Leadership Role: NxStage; LightLine Medical, Home Dialysis University; and Other Interests or Relationships: Section Editor for Dialysis Up To Date; Executive Committee Home Dialysis University.

I understand that the information above will be published within the journal article, if accepted, and that failure to comply and/or to accurately and completely report the potential financial conflicts of interest could lead to the following: 1) Prior to publication, article rejection, or 2) Post-publication, sanctions ranging from, but not limited to, issuing a correction, reporting the inaccurate information to the authors' institution, banning authors from submitting work to ASN journals for varying lengths of time, and/or retraction of the published work.

Name: Thomas A. Golper
Manuscript ID: K360-2025-000465R1
Manuscript Title: Hemodiafiltration should be...... Commentary
Date of Completion: May 1, 2025
Disclosure Updated Date: May 1, 2025
